# S100a9 inhibits Atg9a transcription and participates in suppression of autophagy in cardiomyocytes induced by β_1_-adrenoceptor autoantibodies

**DOI:** 10.1186/s11658-023-00486-1

**Published:** 2023-09-18

**Authors:** Xiaoyan Zhi, Shu Shi, Yang Li, Mingxia Ma, Yaolin Long, Chen Li, Haihu Hao, Huirong Liu, Xiaohui Wang, Li Wang

**Affiliations:** 1 Department of Pathology, Shanxi Medical University, No.56 Xinjian South Road, Taiyuan, Shanxi 030001 People’s Republic of China; 2 Department of Orthopaedics, Shanxi Bethune Hospital, Shanxi Academy of Medical Science, Tongji Shanxi Hospital, Third Hospital of Shanxi Medical University, Taiyuan, People’s Republic of China; 3 Department of Physiology and Pathophysiology, School of Basic Medical Sciences, Capital Medical University, Beijing, People’s Republic of China

**Keywords:** S100a9, β_1_-AA, Autophagy, HIF-1α, Atg9a

## Abstract

**Background:**

Cardiomyocyte death induced by autophagy inhibition is an important cause of cardiac dysfunction. In-depth exploration of its mechanism may help to improve cardiac dysfunction. In our previous study, we found that β_1_-adrenergic receptor autoantibodies (β_1_-AAs) induced a decrease in myocardial autophagy and caused cardiomyocyte death, thus resulting in cardiac dysfunction. Through tandem mass tag (TMT)-based quantitative proteomics, autophagy-related S100a9 protein was found to be significantly upregulated in the myocardial tissue of actively immunized mice. However, whether S100a9 affects the cardiac function in the presence of β_1_-AAs through autophagy and the specific mechanism are currently unclear.

**Methods:**

In this study, the active immunity method was used to establish a β_1_-AA-induced mouse cardiac dysfunction model, and RT-PCR and western blot were used to detect changes in gene and protein expression in cardiomyocytes. We used siRNA to knockdown S100a9 in cardiomyocytes. An autophagy PCR array was performed to screen differentially expressed autophagy-related genes in cells transfected with S100a9 siRNA and negative control siRNA. Cytoplasmic nuclear separation, co-immunoprecipitation (Co-IP), and immunofluorescence were used to detect the binding of S100a9 and hypoxia inducible factor-1α (HIF-1α). Finally, AAV9-S100a9-RNAi was injected into mice via the tail vein to knockdown S100a9 in cardiomyocytes. Cardiac function was detected via ultrasonography.

**Results:**

The results showed that β_1_-AAs induced S100a9 expression. The PCR array indicated that Atg9a changed significantly in S100a9siRNA cells and that β_1_-AAs increased the binding of S100a9 and HIF-1α in cytoplasm. Knockdown of S100a9 significantly improved autophagy levels and cardiac dysfunction.

**Conclusion:**

Our research showed that β_1_-AAs increased S100a9 expression in cardiomyocytes and that S100a9 interacted with HIF-1α, which prevented HIF-1α from entering the nucleus normally, thus inhibiting the transcription of Atg9a. This resulted in autophagy inhibition and cardiac dysfunction.

**Supplementary Information:**

The online version contains supplementary material available at 10.1186/s11658-023-00486-1.

## Introduction

In recent years, the incidence and mortality of cardiac dysfunction have been continually increasing, and as such, it has become an important public health concern [1]. In several studies, cardiomyocyte death has been found to occur during the early stages of cardiac dysfunction, which leads to a progressive loss of cardiomyocytes and even heart failure [2]. Autophagy is an important mechanism for maintaining cellular homeostasis, impaired autophagy contributes to cardiomyocyte cell death [3, 4]. Myocardial autophagy maintains myocardial function by clearing damaged organelles or misfolded proteins [5, 6]. If autophagy is insufficient, it can lead to misfolded proteins and the accumulation of aging cells, leading to the death of cardiomyocytes. Therefore, it is particularly important to clarify the factors that cause autophagic inhibition in cardiomyocytes during the process of cardiac dysfunction.

Researchers have found that β_1_-adrenergic receptor autoantibodies (β_1_-AAs), which combine with β_1_-adrenergic receptor (β_1_-AR), could be detected in the serum of 40–60% of patients with cardiac dysfunction [7]. Overactivation of the β1-adrenergic receptor (β_1_-AR) in cardiomyocytes plays a crucial role in cardiac hypertrophy and its progression to heart failure [8, 9]. Our lab, as well as others, has demonstrated that β_1_-AAs can persistently activate β_1_-AR, which leads to the overactivation of β_1_-AR downstream signaling and cardiac injury, which in turn leads to cardiac dysfunction [10–13]. Removal of serum β_1_-AAs by immunoadsorption significantly improves cardiac function [7, 14, 15], highlighting the importance of β_1_-AAs in cardiac dysfunction. Our research group previously found that β_1_-AAs could significantly reduce the level of myocardial autophagy [13, 16]. Upregulating autophagy in cardiomyocytes with rapamycin effectively improved β_1_-AA-induced cardiomyocyte death and cardiac dysfunction. However, the mechanism underlying β_1_-AA-induced autophagy inhibition in cardiomyocytes is still unclear.

S100a9 is a member of calcium-binding protein family and participates in the pathophysiological processes of inflammatory response and immune regulation. According to the published reports, S100a9 is associated with acute coronary syndrome, atherosclerosis, and heart disease caused by endotoxin [17–21]. In human breast cancer cells, S100a9 could promote autophagy and induce cell death by upregulating the expression of Beclin-1, as well as by promoting the formation of Atg12-Atg5 and lysosomal activation [22]. The main effects of S100a9 in pancreatic cancer are the inhibition of NF-κB and the stimulation of mTOR, which inhibit autophagy [23]. In colon cancer progression, S100a9 is oxidized by NOX1-produced ROS, which facilitates binding to mTORC1 and its activation [24]. mTORC1 inhibits autophagy by binding to the ULK1 complex. S100a9 could also regulate myocardial autophagy through MAPK and PI3K-AKT pathways [25]. However, whether S100a9 plays a role in β_1_-AA-induced autophagy inhibition and the specific mechanism involved are not clear.

Here, we confirmed that β_1_-AAs could increase the expression of S100a9 in cardiomyocytes based on tandem mass tag (TMT) quantitative proteomics. Using adeno-associated virus 9 (AAV9) and small interfering RNA (siRNA) to knockdown myocardial S100a9, we found that S100a9 knockdown could significantly upregulate the decreased myocardial autophagy induced by β_1_-AAs and improve cardiac dysfunction. Moreover, it was further confirmed that upregulation of S100a9 expression could inhibit the entry of hypoxia inducible factor-1α (HIF-1α) into the nucleus by interacting with it. Thus, HIF-1α did not play a role in promoting the transcription of Atg9a and instead inhibited Atg9a expression, thereby also inhibiting the level of autophagy. The novel mechanism of S100a9 inhibiting autophagy can provide an effective therapeutic target for patients with myocardial autophagy disorder.

## Materials and methods

### Experimental animals

C57BL/6 mice (6–8 weeks old, 18–20 g) were purchased from the Experimental Animal Center of Shanxi Medical University. During the entire experiment, all mice were maintained at a temperature of 22–24 °C and a humidity of 40–60%, and they had free access to food and water. All animal procedures conformed to the guidelines of the Ethics Committee of Shanxi Medical University.

### Establishment of the actively immunized mouse model

C57BL/6 male mice (6–8 weeks old) were randomly divided into an active immunization group and a control group. β_1_-AR-ECII, the second extracellular loop of the β_1_-adrenergic receptor (GLS, Shanghai, China), was dissolved in an Na_2_CO_3_ solution and then repeatedly emulsified with complete Freund’s adjuvant (Sigma-Aldrich) until it had the consistency of yogurt. An emulsified and mixed 1:1 solution was injected subcutaneously into the back of the mice at multiple points during the first immunization. Subsequently, incomplete Freund's adjuvant (Sigma-Aldrich) emulsified with β_1_-AR-ECII solution was used to enhance immunity after two weeks. An Na_2_CO_3_ solution was used as the solvent control group [13].

### Animal grouping and treatment

Male C57BL/6 mice (6–8 weeks old) were divided into the following groups: (1) an AAV9-Vector-RNAi group; (2) an AAV9-vector-RNAi + β_1_-AA group; (3) an AAV9-S100a9-RNAi group; and (4) an AAV9-S100a9-RNAi + β_1_-AA group. The mice were injected with AAV9-NC and AAV9-S100a9-RNAi knockdown virus, and 5 × 10^11^ genome copies (vg)/mouse were delivered via the tail vein. AAV9 carrying S100a9 RNAi was synthesized by Genechem Co., Ltd (Shanghai, China). Five weeks after virus delivery, an S100a9 knockdown effect was detected and active immunization was performed.

### Streptavidin (SA)- ELISA

The level of β_1_-AA was detected as described in previous studies [13, 26]. β_1_-AR-ECII was dissolved in Na_2_CO_3_ to prepare a 10 μg/ml final concentration solution. Then, 50 μl of antigen was added to a microtiter plate at 4 °C overnight for 12–16 h, at which point it became a solid antigen. The plate was blocked for 1 h with 5% milk, and then the tested serum was diluted with blocking solution at a volume ratio of 1:10 and added to 96-well plate, after which it was incubated for 1 h at 37 °C. Next, biotinylated secondary antibody and horseradish peroxidase streptavidin (SA-50004, Burlingame, USA, 1:3000) were added to the plate, and then it was incubated for 1 h at 37 °C. Then, 2, 2′-azinodi (ethylbenzthiazoline) sulfuric salt (ABTS, Bio Basic Inc., AD0002, Markham, ON, Canada) was dissolved in a substrate containing citric acid and Na_2_HPO_4_, after which 3% H_2_O_2_ was added at a volume ratio of 1:2000 and mixed well. A total of 50 μl of the resulting solution was then added into each well, and the plate was incubated in the dark for 30 min. Finally, OD values were measured at 405 nm.

### Affinity chromatography

An actively immunized mice model was established as in previous studies [13]. The sera from rats were collected, and then a MAbTrap kit (GE Healthcare, Uppsala, Sweden) was used to extract and purify IgG. Triple-distilled water was used to elute the ethanol in the chromatographic column, and the chromatographic column was moistened with binding buffer. Then, the rat serum was filtered slowly through the chromatographic column to fully combine the antigen and antibody. Binding buffer was used to wash the nonspecific binding antibodies. Elution buffer was then added to the chromatographic column and the antibody was washed out. The elution liquid was collected into a tube and concentrated with a concentration filter. Next, the concentration was detected using a bicinchoninic acid (BCA) kit (Boster AR0146) and stored at − 80 °C for later use. Binding buffer was added to wash the column, after which ethanol was reinjected into the chromatographic column and stored at 4 °C.

### Quantitative proteomics

The hearts of mice successfully immunized were harvested and sent to the Jingjie PTM Biolab Co., Ltd. (Hang Zhou, China) for proteomic analysis. Samples were removed from their − 80 °C storage, lysis buffer was added to them, and then they were sonicated. After centrifugation, the supernatant was collected to detect protein concentration using a BCA kit. Each sample was subjected to enzymolysis. Tryptase was added for enzymolysis overnight, and then the peptide fragments were labeled with TMT. Finally, the samples underwent high-pH reversed-phase high-performance liquid chromatography (HPLC) and liquid chromatography-mass spectrometry analysis [27].

### Small animal ultrasound

A small animal ultrasound can reflect the cardiac function of mice. Gaseous anesthesia was administered to the mice, and then the mice were placed in anesthesia boxes. The anesthetized mice were fixed on the plate, and were then depilated in the left chest position. The mice’s cardiac function was examined via M-mode Teichholz ultrasound. Short-axis sections were taken [26].

### Cell culture and stimulation

H9c2 cells (GNR 5) were purchased from the Chinese Academy of Sciences Cell Bank (Shanghai, China). H9c2 cells were cultured in Dulbecco’s Modified Eagle Medium (DMEM, Gibco) supplemented with 10% fetal bovine serum (FBS, Cellmax, Beijing, China). Penicillin and streptomycin were added to the cells at a ratio of 1:100, after which they were incubated at 37 °C and 5% CO_2_. Cells were subcultured until the culture reached 80% density. The cell lines were transfected with siRNAs (sequences shown in Additional file 1: Table S2) and plasmids using RNAFit or Lipofectamine 3000, according to the manufacturer’s instructions.

### Antibodies, plasmids, and siRNAs

LC3B (ab48394, Abcam, 1:1000), p62 (ab56416, Abcam, 1:1000), S100a9 (ab242945, Abcam, 1:1000), HIF-1ɑ (CPA9305, Cohesion Biosciences), Atg9a (ab108338, Abcam), β-actin (81115-1-RR, Proteintech), and GAPDH (bsm-33033M, Bioss) were used for western blot. S100a9 (26992-1-AP, Proteintech) and HIF-1ɑ (sc-13515, Santa Cruz) were used for immunofluorescence.

### RNA interference

RNAFit (Hanbio Co., Ltd., Shanghai, China) was used for S100a9 knockdown. H9c2 cardiomyocytes were incubated in a 6-well plate until the density was close to 80%. The cells were washed and transiently transfected with siRNAs (100 nM) using RNAFit and then divided into siRNA-S100a9 and negative siRNA control groups. Next, 10 μl of siRNA was added to the 200-μl Opti-MEM and gently mixed 3–5 times with a pipette gun. Then, 30 μl of RNAFit was added to the mixture and vortexed for 10 s. It was then incubated at room temperature for 10 min so that the siRNA and RNAFit formed a transfection complex. At the same time, the original culture solution was removed and replaced with 1.8 ml of fresh DMEM. Then, the abovementioned transfected compound was added to the plate, and the plate was gently shaken. The final volume of culture medium in each well was 2 ml, and the final concentration of siRNA was 100 nM. After transfection for 6 h, it was considered to have a sufficiently high transfection efficiency. At this point, the original medium was discarded and the cells were given fresh medium supplemented with 10% FBS.

### Plasmid transfection

LipoFiter3.0 Liposomal (Hanbio Co., Ltd., Shanghai, China) was used for plasmid transfection. H9c2 cells were transfected with S100a9 and Atg9a plasmids using Lip3.0. Plasmid (4 μg) was added to a sterile tube containing 250 μl of DMEM and gently mixed in. A total of 250 μl of DMEM and 6 μl of LipoFiter3.0 was then added to another clean sterile tube and incubated at room temperature for 5 min. The DNA solution and the LipoFiter3.0 solution were then mixed and incubated for 20 min, after which the LipoFiter3.0–DNA mixture was added to the plate. After 6 h of transfection, the serum-free medium containing LipoFiter 3.0 -DNA was discarded. A total of 2 ml of cell culture medium containing 10% FBS was added to the medium. At 48 h post-transfection, samples were collected for the next experiment.

### Western blot

Samples were collected and added to radioimmunoprecipitation assay (RIPA) buffer (AR102-100, BOSTER) containing 1% protease inhibitor (AR1178, BOSTER) and phosphate inhibitor (AR1195, BOSTER) for protein extraction. Subsequently, ultrasonic treatment was performed. The ultrasonic conditions were as follows: the output electric power was 30–40%, every ultrasound lasted for 10–15 s each time, the entire treatment was performed three times (with an interval of 15 s between each ultrasound), and the current was about 30 A. After centrifugation, the supernatant was collected and the protein concentration was measured. Then, proteins were isolated using SDS-PAGE gel, followed by protein transfer. After this, the membrane was blocked with 0.5% milk powder and the specific primary antibody was incubated 12–16 h at 4 °C. Secondary antibodies were incubated at 4 °C for 2 h. The membrane was then visualized using chemiluminescence methods. Images were acquired using the Bio-Rad microimaging system and analyzed using Image J.

### RT-PCR

Total RNA was extract using Trizol (Takara, JPN), and then a spectrophotometer was used to measure RNA concentration. The samples were quantified to 1 μg and reverse transcribed with a PrimeScript RT kit (Takara, RR047A, JPN). Quantitative PCR was conducted using an RT-qPCR™ rapid qPCR hybrid kit with TB Green (Takara, RR430A, JPN). The experiment was repeated at least five times (biological replicates). The primer sequences are shown in Additional file 1: Table S3.

### PCR-array

All PCR reactions were carried out using a VII Atm7 Real-time PCR system (WC Gene Biotechnology, Shanghai). All samples contained 91 autophagy-related genes. The cells were lysed with Trizol, while chloroform and isopropanol were used to extract RNA. The qPCR mixture (10 μl) was composed of 5 μl of Roche FastStart Universal SYBR Green Master (2×), 0.75 μl of each primer (10 μM), 3 μl of ddH20, and 0.5 μl of the template. Initial enzyme activation was performed at 95 °C for 10 min, followed by 40 cycles of denaturation at 95 °C for 30 s and annealing at 60 °C for 30 s. A melting curve ranging from 60 °C to 95 °C was generated to determine the specificity of amplification [28, 29]. Data with a fold change (FC) > 2 or < 0.8 and *P* < 0.05 were considered statistically significant.

### Co-IP experiment

ProteinA/G magnetic beads were incubated with S100a9 antibody, HIF-1α antibody, and rabbit IgG antibody at 4 °C for 6 h. Then, bead–antibody complexes were washed three times, after which the complexes were incubated with the collected protein lysates overnight. The beads were washed three times. Then, 5 × loading buffer was added, boiled for 10 min to denature them, and then detected via western blot.

### Nuclear-cytoplasmic separation

The control group and β_1_-AA group cells were collected on ice with a scraper. The cells were centrifuged for 2–3 min and the supernatant was removed. Then, reagent A, containing phenylmethanesulfonyl fluoride (PMSF), was added to the centrifuge tube and incubated on ice for 10 min, after which it was centrifuged for 10 min at 4 °C. Next, the supernatant was collected in a pre-cooled centrifuge tube and the cytoplasmic protein was obtained. The precipitate was collected for nucleoprotein extraction and was incubated on ice for 10 min with nucleoprotein extraction reagent B. Then, the precipitate was centrifuged for 10 min. The supernatant was collected in precooled tubes and the extracted nuclear protein was obtained. The protein concentration was measured using a BCA kit.

### Immunofluorescence

Cells were fixed in 4% paraformaldehyde for 15 min and then washed three times. The cell slides were transferred sequentially to 0.5% TritonX-100 for 15 min and 5% BSA solutions for 30 min. The solution was then discarded and the cells were immediately incubated with S100a9 and HIF-1α antibodies for 16 h at 4 °C. The sections were washed three times and incubated with fluorescent secondary antibodies at 37 °C for 1 h in the dark. The slides were sealed with mounting medium containing DAPI. Immunofluorescence images were obtained with a laser scanning confocal microscope.

### Dual-luciferase reporter assay

When the cell density reached 1 × 10^5^ cells/well, the cells were transfected with luciferase constructs either with or without HIF-1α plasmids. After transfection for 24 h, the medium was discarded. The cells were washed gently, and cell lysis buffer was added to the cells for 15 min. All of the cell lysate was collected into a tube and then centrifuged at 16,000 rpm for 5 min, after which the supernatant was collected for subsequent detection. A total of 20 μl of cell lysis supernatant was then added into a detection tube, followed by 100 μl of firefly luciferase reaction buffer containing a substrate balanced to room temperature, and the activity of luciferase was immediately detected using a Dual-Luciferase Reporter Assay System according to the manufacturer’s instructions (Molecular Devices, USA). Then, 100 μl of Renilla substrate was added to detect the Renilla activity. A Luciferase Reporter Assay kit (MA0518; Dalian Meilun Biotechnology) was used to detect the luciferase activity.

### Statistical analysis

Statistical analysis was performed with SPSS 16.0 software. Statistical significance was determined by one-way ANOVA followed by Kruskal–Wallis multiple-comparison tests or two-way ANOVA followed by Dunn post-tests. A *p* < 0.05 was considered to indicate statistical significance. All data were expressed as the mean ± SEM.

## Results

### Mass spectrometry-based quantitative proteomics analysis of mouse heart treated with β_1_-AAs

**Fig. 1:**
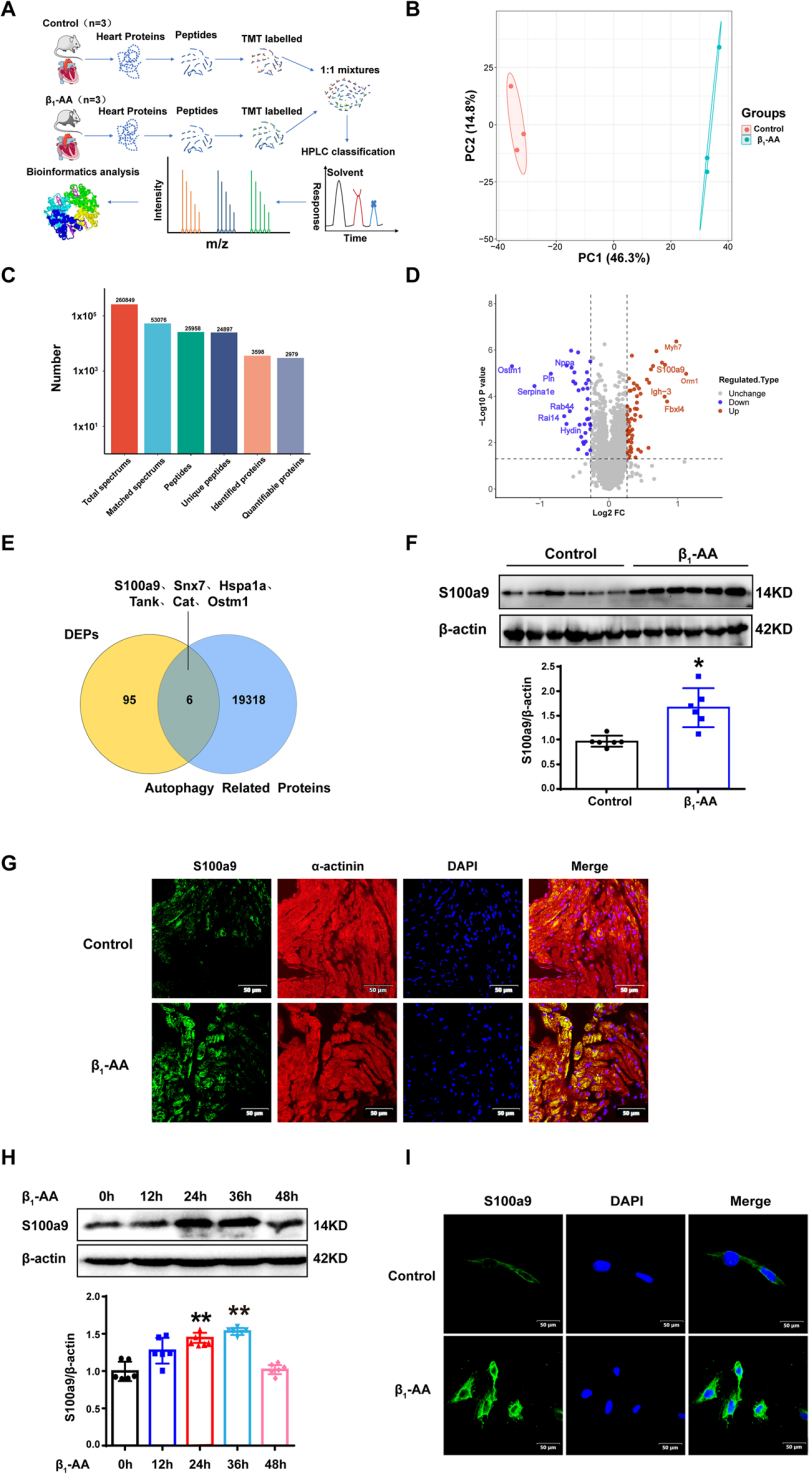
Proteomics showed that S100a9 was the main mediator underlying the decrease of myocardial autophagic flux. **A** Flowcharts of the quantitative proteomics performed in the present study. **B** The principal component analysis (PCA) of proteins by proteomics. **C** Number of identified peptide segments and proteins in the database after filtering. **D** Volcano plot of the proteins in the myocardial tissues of mice. The red dots indicate upregulated proteins, and the blue dots indicate downregulated proteins. **E** Venn diagram of the statistically significant proteins and autophagy-related proteins. **F** S100a9 expression levels were analyzed via western blot. **G** Mouse myocardial tissue sections were stained via immunofluorescence to detect the expression of S100a9 in cardiomyocytes. Representative immunofluorescence images showing α-actinin (red), S100a9 (green), and DAPI (blue). **H** S100a9 expression levels were detected after β_1_-AA stimulation for 12, 24, 36, 48 h in H9c2 cells. **I** S100a9 expression levels were detected via immunofluorescence staining after β_1_-AA stimulation for 24 h in H9c2 cells. S100a9 was stained with FITC (scale bar, 50 µm). Results are presented as the mean ± SEM with *n* = 6 per group. *p < 0.05 vs. control, **p < 0.01 vs. control. DEPs indicate differentially expressed proteins

In this study, differential protein expression in the myocardial tissue of actively immunized mice was detected by TMT-based quantitative proteomics (Fig. 1A). The results of protein quantitative principal component analysis of all samples showed good quantitative repeatability (Fig. 1B). There were 260,849 secondary spectra obtained through mass spectrometric detection. Based on the analysis of the MS results, 25,958 peptides were identified in total, with 24,897 unique peptides. Among them, 3598 protein species were identified, of which, 2,979 were quantified (Fig. 1C), and quantitative analysis of MS data met the quality control criteria (Additional file 1: Figure S1). The proteins with fold change > 1.2 or < 0.8 and *p* < 0.05 were considered significantly changed proteins. Compared with the control group, high-throughput proteomics analysis identified 103 different proteins, 65 proteins were significantly upregulated, whereas 38 proteins were found to be downregulated (Fig. 1D and Additional file 1: Figure S2). Further, to screen the proteins associated with autophagy, we acquired six overlapping proteins by intersecting the proteomic and autophagy datasets, and S100a9 was the top upregulated protein (Fig. 1E, Additional file 1: Table S1). These results indicated that upregulation of S100a9 might be an important reason for the decreased autophagy induced by β_1_-AAs in cardiomyocytes.

To further verify the results of proteomics, we established a β_1_-AA positive mouse model using an active immunity method and detected the serum β_1_-AA level by ELISA. The results showed that the OD value of serum β_1_-AAs in immunized mice was increased significantly (Additional file 1: Figure S3), suggesting that the model was successfully established. Western blot analysis was used to determine the S100a9 levels in the myocardium after 4 weeks of active immunization. The results showed that S100a9 was significantly increased (Fig. 1F). Further, S100a9 was observed by immunofluorescence staining. α-actinin was used to label cardiomyocytes, and the results indicated that the green fluorescence representing S100a9 in the myocardial tissue was significantly increased and co-localized with cardiomyocytes (Fig. 1G).

Then, H9c2 cells were treated with 1 μmol/L β_1_-AAs at different time points. Western blot results showed that S100a9 expression was increased at 24 h and 36 h after β_1_-AA stimulation (Fig. 1H). Further, immunofluorescence staining was used to show that the green fluorescence representing S100a9 was weakly expressed in the control group, and the green fluorescence was increased significantly after 24 h of β_1_-AA stimulation (Fig. 1I). The above results indicated that β_1_-AA could significantly increase the expression of S100a9 in cardiomyocytes.

### Upregulation of S100a9 expression inhibited the autophagic flux induced by β_1_-AAs

**Fig. 2:**
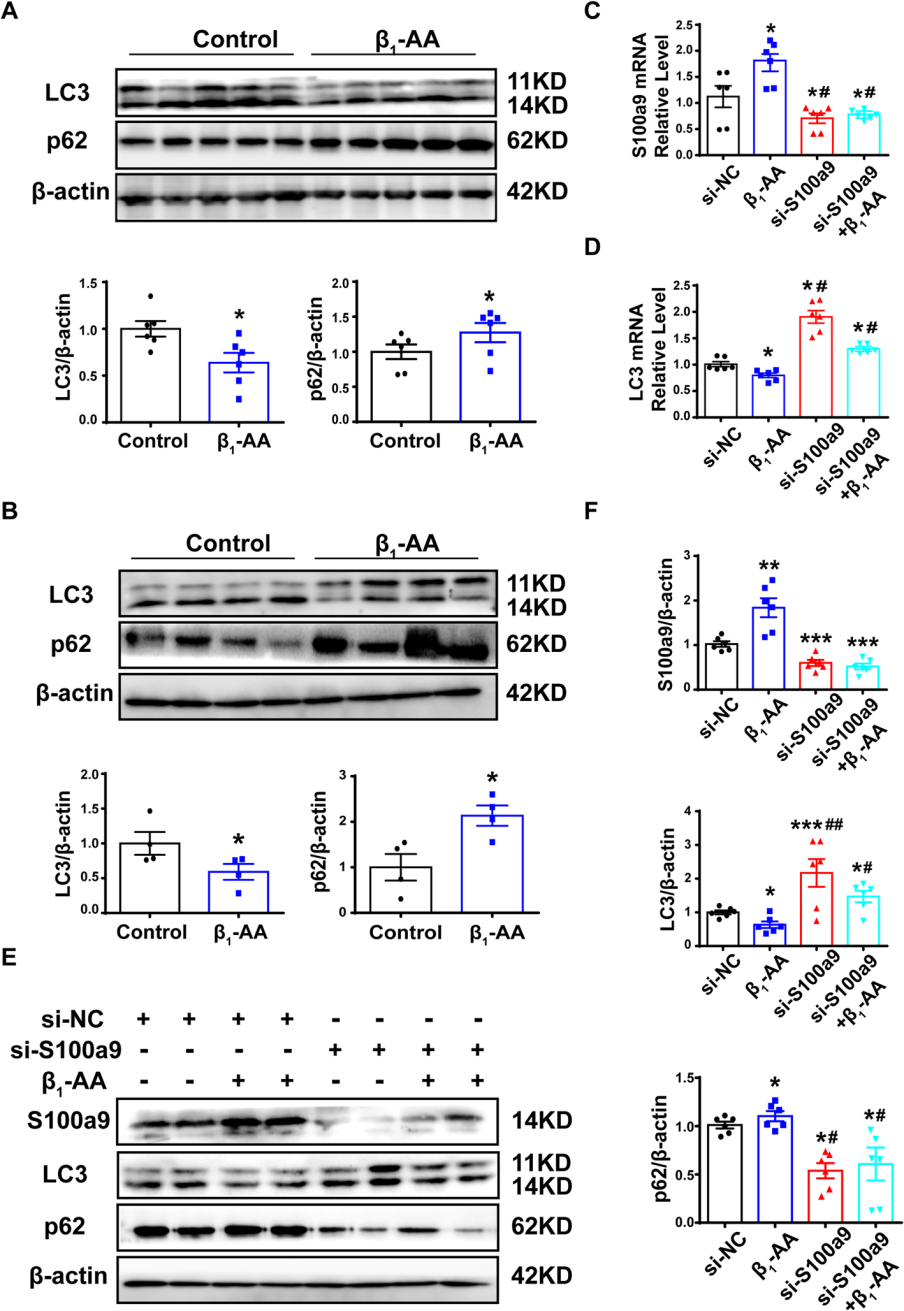
Upregulation of S100a9 expression participates in β_1_-AA-induced autophagy inhibition in cardiomyocytes. **A** LC3 and p62 protein expression in C57BL/6 mice actively immunized for 4 weeks were detected by western blot (*n* = 6 per group). **B** The levels of LC3 and p62 in cardiomyocytes stimulated with β_1_-AAs were detected by western blot (*n* = 4 per group). **C**, **D** The mRNA levels of S100a9 and LC3 in H9c2 cells stimulated with β_1_-AAs after transfection with S100a9 siRNA or NC siRNA were detected by RT-PCR (*n* = 6 per group). **E** S100a9, LC3, and p62 protein expression in H9c2 cells treated with β_1_-AAs after transfected with S100a9 siRNA or NC siRNA were detected by western blot (*n* = 6 per group). **F** Statistical diagram of S100a9, LC3, and p62. Data are presented as the mean ± SEM; *p < 0.05 vs. control, **p < 0.01 vs. control, ^#^*p* < 0.05 vs. β_1_-AA group, ^##^*p* < 0.01 vs. β_1_-AA group

To observe the effect of β_1_-AAs on the autophagy of cardiomyocytes, the autophagy markers LC3 and P62 in cardiomyocytes were detected. The results showed that LC3 protein in actively immunized myocardial tissue was significantly decreased, and P62 expression was significantly increased (Fig. 2A). After β_1_-AA treatment of H9c2 cells for 24 h, LC3 expression was decreased, while P62 was increased. Thus, the results suggested that β_1_-AAs could lead to a decrease in autophagic flux in cardiomyocytes (Fig. 2B).

To further clarify the effect of S100a9 upregulation on myocardial autophagy, siRNA was used to knockdown S100a9 in cardiomyocytes. Three different siRNAs targeting S100a9 were designed to silence S100a9 expression, and the knockdown effects were detected. The results showed that Sequences 1 and 3 caused significant knockdown effects, while Sequence 2 did not (Additional file 1: Figure S4, Table S2). Therefore, Sequence 1, with the highest knockdown effect, was selected for subsequent experiments. Knocking down S100a9 resulted in an increase in LC3, but a decrease in p62, as compared with the control group (Additional file 1: Figure S5), suggesting that S100a9 itself had the effect of inhibiting autophagy in cardiomyocytes. Furthermore, cardiomyocytes with S100a9 knockdown were treated with β_1_-AAs. RT-PCR results showed that S100a9-knockdown could significantly improve β_1_-AA-induced decrease in the LC3 mRNA level (Fig. 2C, D). Western blot analysis confirmed that S100a9-knockdown could significantly reverse β_1_-AA-induced decrease in LC3 protein level and p62 protein accumulation (Fig. 2E, F). These results suggested that upregulation of S100a9 expression induced by β_1_-AA was an important factor for autophagy suppression in cardiomyocytes.

### β_1_-AA-induced upregulation of S100a9 led to suppression of cardiomyocyte autophagy via inhibition of Atg9a transcription

**Fig. 3:**
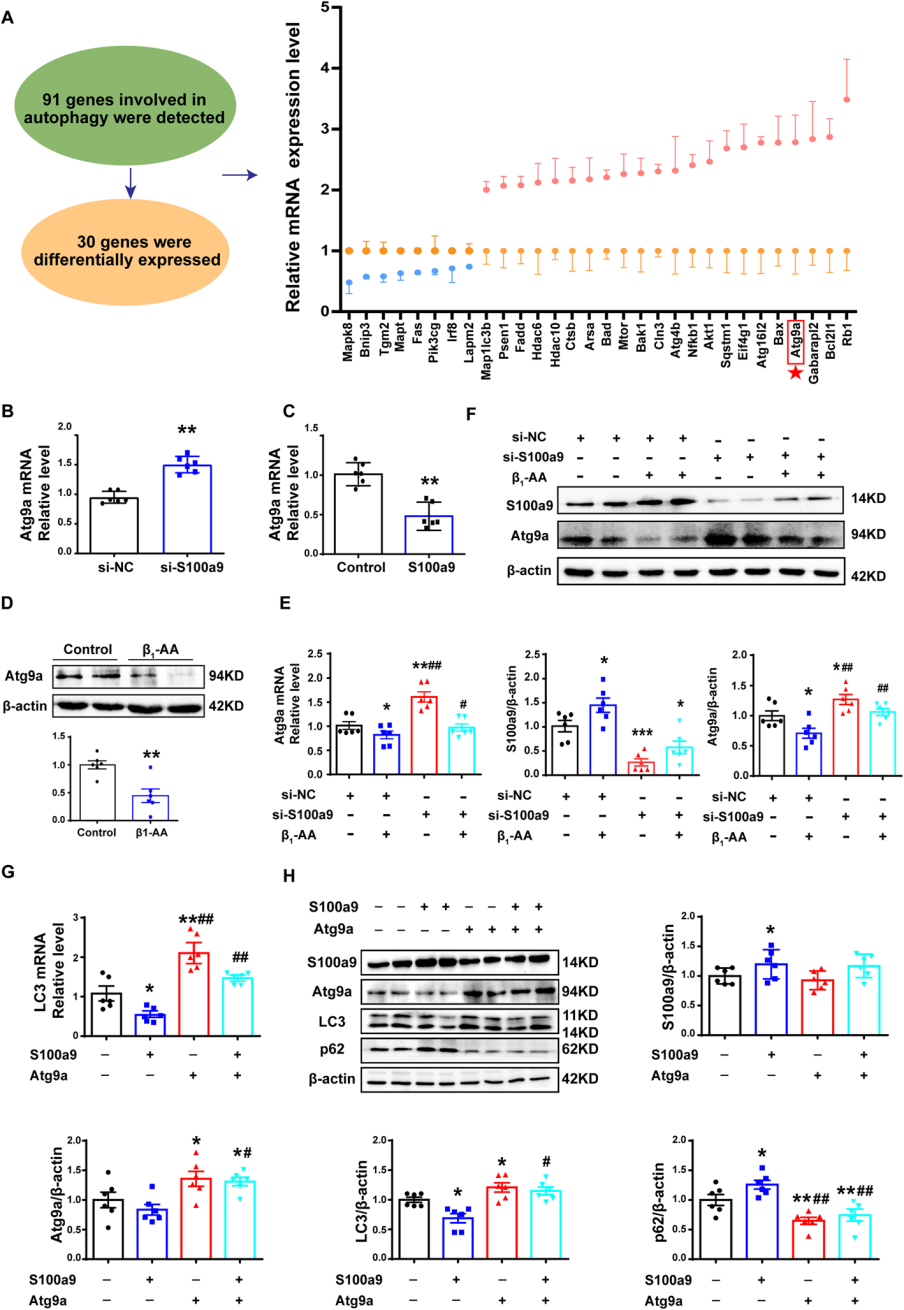
Upregulation of S100a9 inhibits Atg9a transcription, which leads to inhibition of autophagy in cardiomyocytes. **A** Relative mRNA expression levels of 30 autophagy-related genes in cardiomyocytes. **B** Atg9a mRNA was assayed by RT-PCR after transfection with S100a9 siRNA or NC siRNA. **C** Atg9a mRNA was detected by RT-PCR after transfection with the S100a9 plasmid. **D** Atg9a protein expression was measured by western blot. **E** Atg9a mRNA in H9c2 cells treated with β_1_-AAs after transfection with S100a9 siRNA or NC siRNA was assayed by RT-PCR. **F** Western blot analysis for determining the level of Atg9a in H9c2 cells treated with β_1_-AAs after transfection with S100a9 siRNA or NC siRNA. **G** The expression level of LC3 in cardiomyocytes transfected with the S100a9 plasmid and Atg9a plasmid was detected by RT-PCR. **H** LC3 and p62 protein expression in H9c2 cells transfected with an S100a9 plasmid and Atg9a plasmid was detected by western blot. Cardiomyocytes were stimulated with β_1_-AAs for 24 h. Data are presented as the mean ± SEM, with *n* = 6 per group; *p < 0.05 vs. control, **p < 0.01 vs. control. **E** and **F** ^#^*p* < 0.05 vs. β_1_-AA group, ^##^*p* < 0.01 vs. β_1_-AA group. **G** and **H** ^#^*p* < 0.05 vs. S100a9 overexpression group, ^##^*p* < 0.01 vs. S100a9 overexpression group

To further explore the mechanism of S100a9 inhibiting cardiomyocyte autophagy, we used PCR array to detect the mRNA levels of 91 autophagy-related genes in cardiomyocytes following knockdown of S100a9. Genes with FC > 2 or < 0.8 and *p* < 0.05 were considered significantly changed genes. The results showed that 22 genes were upregulated and 8 genes were downregulated in siRNA-S100a9-treated cardiomyocytes (Additional file 2). Among them, Atg9a mRNA expression was dramatically increased (Fig. 3A, Additional file 1: Figure S6). To validate the results of the PCR array, we further detected the Atg9a mRNA level and found that knockdown of S100a9 indeed increased the mRNA level of Atg9a (Fig. 3B), while overexpression of S100a9 significantly decreased the mRNA level of Atg9a (Fig. 3C). The above results indicated that S100a9 could restrain autophagy by inhibiting Atg9a transcription. Furthermore, we observed the effect of β_1_-AAs on the expression of Atg9a and found that β_1_-AAs could decrease the Atg9a mRNA and protein levels in cardiomyocytes (Fig. 3D, E), while knockdown of S100a9 could significantly ameliorate the decrease in Atg9a mRNA and protein levels induced by β_1_-AAs (Fig. 3E, F). To further verify whether Atg9a was the key molecule in the autophagy inhibition induced by S100a9 upregulation, the expression levels of LC3 and p62 were detected. The results showed that Atg9a overexpression could reverse the decrease in LC3 and increase in p62 induced by S100a9 overexpression (Fig. 3G, H). Therefore, these results indicated that S100a9 inhibited autophagy via Atg9a expression in cardiomyocytes.

### β_1_-AA impaired the transcriptional activity of HIF-1α protein target Atg9a in cardiomyocytes

**Fig. 4:**
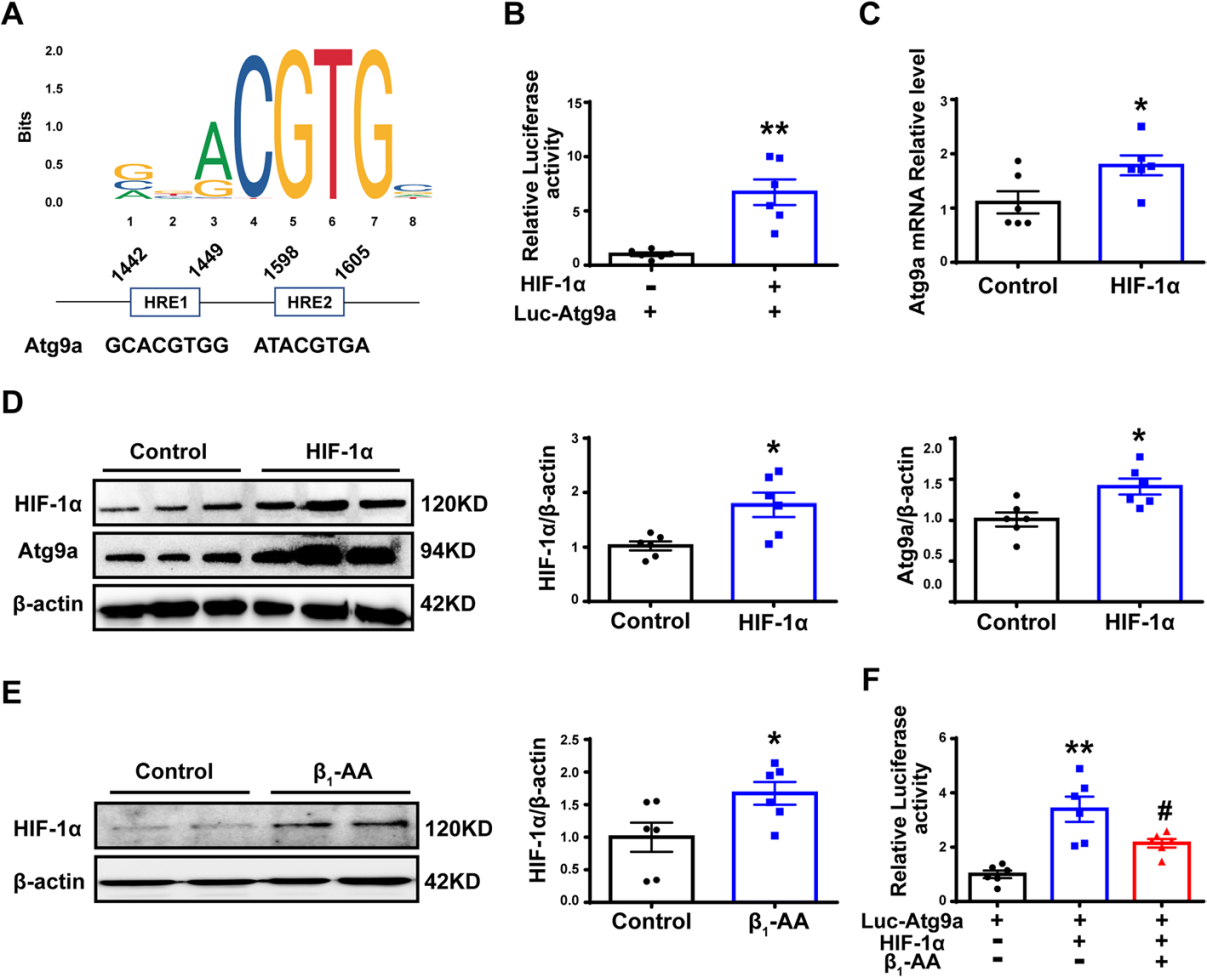
β_1_-AA-induced upregulation of HIF-1α did not promote transcription of Atg9a. **A** JASPAR analysis (http://jaspar.genereg.net/) revealed the recognition sites of HIF-1α on the promoter sequence of Atg9a. **B** Detection of luciferase activity after the Atg9a promoter sequence plasmid and HIF-1α plasmid were transfected into H9c2 cells. **C** Atg9a mRNA level was assayed by RT-PCR. D) Atg9a protein expression was detected by western blot. **E** Western blot analysis for determining the HIF-1α level after β_1_-AA treatment in whole cell lysates. **F** Double-luciferase reporter gene assay of cardiomyocytes treated with β_1_-AAs was used to detect the luciferase activity of Atg9a promoter. Data are presented as the mean ± SEM with *n* = 6 per group; *p < 0.05 vs. control, **p < 0.01 vs. control, and ^#^*p* < 0.05 vs. Luc-Atg9a + HIF-1α group

To further explore the factor that caused the reduced transcription of Atg9a, we predicted the upstream transcription factor of Atg9a through the JASPAR database (http://jaspar.genereg.net/). We found that HIF-1α was one of the transcription factors of Atg9a with a predicted binding site on the promoter sequence of Atg9a (Fig. 4A). We constructed a luciferase reporter of Atg9a. Overexpression of HIF-1α increased the luciferase activity of Atg9a, suggesting that HIF-1α can bind to the promoter of Atg9a to promote transcription (Fig. 4B). Furthermore, when a HIF-1α plasmid was transfected into H9c2 cells, both the transcription and protein levels of Atg9a were significantly increased (Fig. 4C, D), suggesting that HIF-1α could act as a transcription factor of Atg9a. Moreover, we also observed the effect of β_1_-AAs on HIF-1α in cardiomyocytes, and results showed that β_1_-AAs could induce HIF-1α expression in H9c2 cells (Fig. 4E). However, the double-luciferase reporter gene assay showed that after β_1_-AAs were applied to cardiomyocytes overexpressing HIF-1α, the luciferase activity reflecting Atg9a transcription was decreased significantly (Fig. 4F), suggesting that β_1_-AAs could inhibit the transcription-promotion effect of HIF-1α on Atg9a in cardiomyocytes.

### β_1_-AA-induced upregulation of S100a9 combined with HIF-1α to prevent HIF-1α from entering the nucleus

**Fig. 5:**
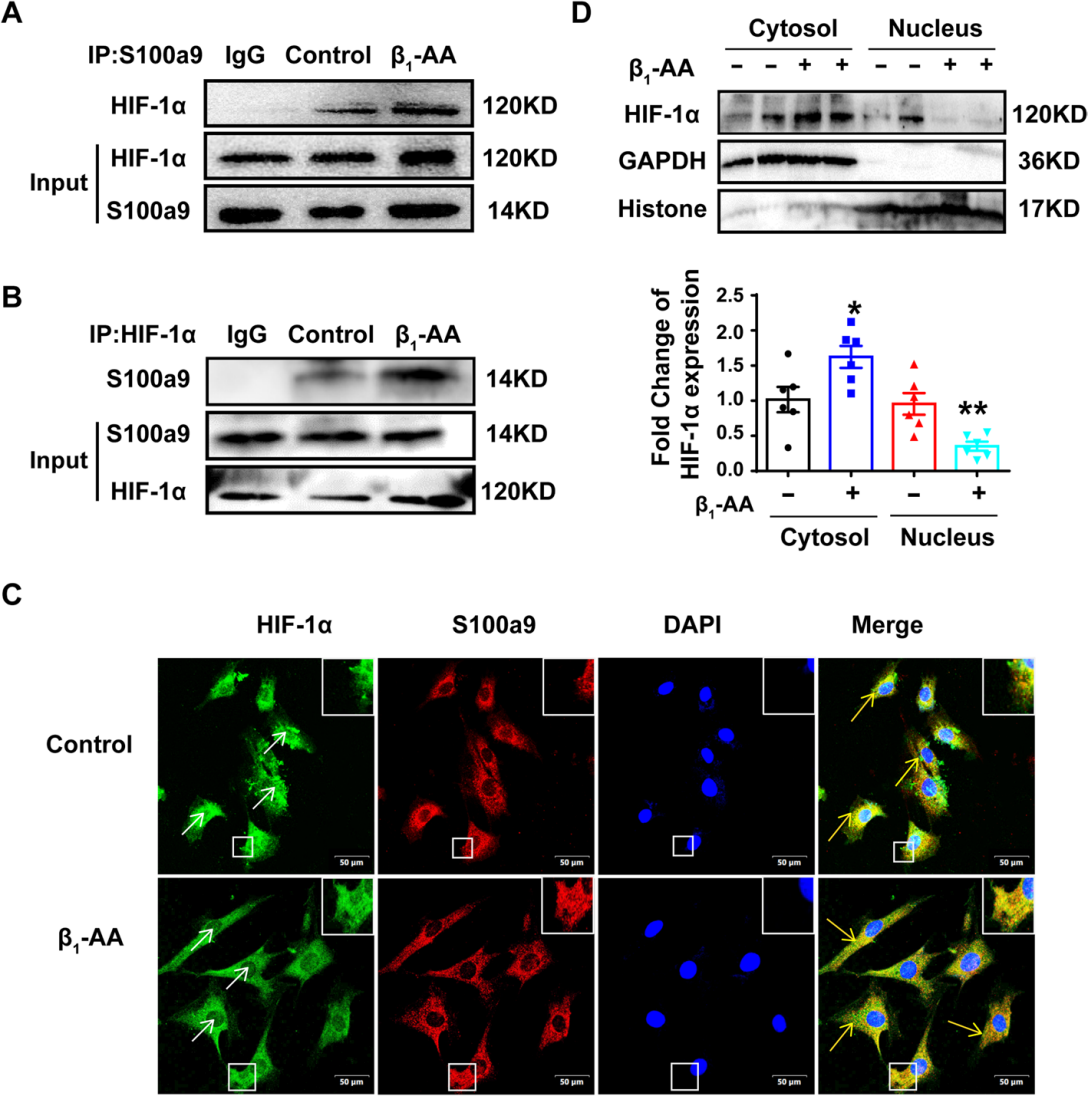
Combination of S100a9 and HIF-1α prevented HIF-1α from entering the nucleus. **A**, **B** Immunoprecipitation showed the interaction between S100a9 and HIF-1α in cardiomyocytes treated with β_1_-AAs for 24 h. **C** Immunofluorescence was used to detect the nuclear translocation and cytoplasmic co-localization of HIF-1α after β_1_-AAs administration for 24 h via immunofluorescence. The white arrows indicate HIF-1α protein in the nucleus. The yellow arrows indicate cytoplasmic co-localization of the HIF-1α protein and S100a9. **D** The distribution of HIF-1α in the cytoplasm and nucleus in cardiomyocytes incubated with β_1_-AAs for 24 h was detected. Data are presented as the mean ± SEM with *n* = 6 per group; *p < 0.05 vs. control, **p < 0.01 vs. control

To explore how HIF-1α upregulation induced by β_1_-AAs failed to promote Atg9a transcription, a co-immunoprecipitation (Co-IP) was conducted to detect the interaction of S100a9 with HIF-1α. The results showed that S100a9 binding with HIF-1α was increased in cardiomyocytes after β_1_-AA treatment (Fig. 5A, B). Immunofluorescent double staining also revealed that β_1_-AAs could significantly increase the green fluorescence of HIF-1α in the cytoplasm of cardiomyocytes, and the co-localization of S100a9 and HIF-1α in the cytoplasm of cardiomyocytes was increased (Fig. 5C). Therefore, we speculated that S100a9 can interact with HIF-1α to inhibit its entry into the nucleus, thereby inhibiting Atg9a expression. To further confirm this conjecture, we conducted nuclear and cytoplasmic fractionation experiments in H9c2 cells. The results showed that β_1_-AAs significantly prevented HIF-1α translocation into the nucleus (Fig. 5D). These findings indicated that β_1_-AAs could cause increased binding of S100a9 and HIF-1α in the cytoplasm of cardiomyocytes, so that HIF-1α could not enter the nucleus to promote the transcription of Atg9a.

### S100a9 knockdown significantly improved β_1_-AA-induced cardiac dysfunction and decreased autophagy in mice

**Fig. 6:**
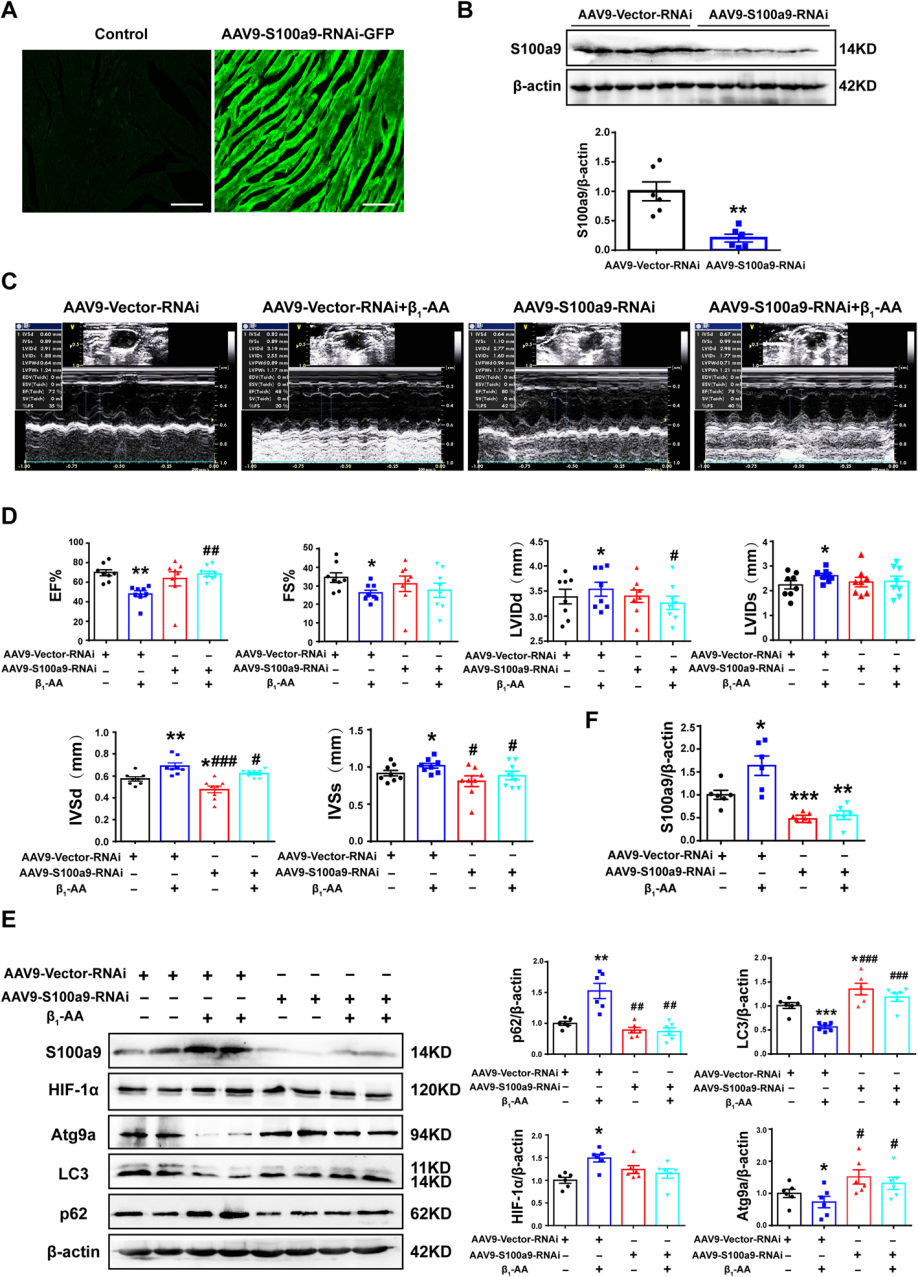
AAV9-S100a9-RNAi improved β_1_-AA-induced cardiac dysfunction and decrease in myocardial autophagic flux in mice. **A** Fluorescence detection of virus enrichment in frozen sections of myocardium. **B** Western blot analysis was used to determine the level of S100a9 in the myocardial tissue of the AAV9-Vector-RNAi group and AAV9-S100a9-RNAi group. **C** M-mode echocardiography images of immunized mice. **D** A small animal ultrasound was used to detect the EF%, FS%, LVIDd, LVIDs, IVSd, and IVSs (*n* = 8 per group). **E** Western blot analysis was used to determine the levels of S100a9, HIF-1α, Atg9a, LC3, and p62 (*n* = 6 per group). **F** Statistical chart of the expression levels of S100a9, HIF-1α, Atg9a, LC3, and p62 in the myocardial tissue. Data are presented as the mean ± SEM; *p < 0.05 vs. control, **p < 0.01 vs. control, and ^#^*p* < 0.05 vs. β_1_-AA group, ^##^*p* < 0.01 vs. β_1_-AA group

To confirm whether S100a9 knockdown could improve β_1_-AA-induced cardiac dysfunction in mice, we injected AAV9-S100a9-RNAi into mice via the tail vein to knockdown S100a9 in cardiomyocytes (Additional file 1: Figure S7). AAV9-GFP expression showed enrichment of green fluorescence in mouse heart 12 weeks after intravenous tail injection (Fig. 6A), suggesting that the virus infected the heart successfully. Western blot analysis showed that AAV9-S100a9-RNAi successfully knocked-down S100a9 (Fig. 6B). Then, the mice were actively immunized for 12 weeks, and the cardiac function was detected by mouse ultrasonography. The left ventricular ejection fraction (EF%) and fractional shortening (FS%), reflecting myocardial contractility, were significantly decreased in the actively immunized mice. The left ventricular end-diastolic diameter (LVIDd) and the left ventricular end-systolic diameter (LVIDs), reflecting myocardial diastolic capability, were significantly increased. The interventricular septal thickness at diastole (IVSd) and the interventricular septal thickness at systole (IVSs), reflecting the left ventricular wall thickness, were also significantly increased. S100a9 knockout in cardiomyocytes could significantly reverse β_1_-AA-induced reduced EF% and increased LVIDd, IVSd, and IVSs. But AAV9-S100a9-RNAi could not reverse the decrease in FS% and the increase in LVIDs induced by β1-AA (Fig. 6C, D; Additional file 3). Western blot analysis also showed that knockdown of S100a9 significantly reversed the decreased autophagy induced by β_1_-AAs (Fig. 6E, F). The above results indicated that knockdown of S100a9 could significantly improve autophagy levels and cardiac dysfunction.

## Discussion

The death of cardiomyocytes induced by autophagy inhibition is an important reason for the progression of cardiac dysfunction [13, 30]. Autophagy is an important mechanism for maintaining cell homeostasis via the degradation of damaged, aged, or excess biological macromolecules and organelles [3]. The optimal window of autophagy activity is crucial for maintaining cardiomyocyte homeostasis and function. Autophagy deficiency leads to the accumulation of incorrectly folded proteins and aging cells, which collectively induce cardiomyocyte death and trigger heart disease [31, 32]. Therefore, it is particularly crucial to identify the factors that cause autophagy inhibition in cardiomyocytes during the process of cardiac dysfunction. In our previous research, we demonstrated that β_1_-AAs inhibited autophagy through activating the β_1_-AR/cAMP/PKA signaling pathway [13]. In this research, we established a β_1_-AA-immunized mouse model of cardiac dysfunction and the corresponding cardiomyocyte model. The results showed that β_1_-AAs could lead to significantly decreased autophagy in cardiomyocytes, manifested as a decrease in the autophagy marker protein LC3 and an increase in autophagy substrate p62 protein, which is consistent with our previous study [13, 16].

To further explore the specific mechanism of β_1_-AA-induced autophagy suppression, we performed a label-free quantitative proteomic study and identified 65 proteins that were significantly upregulated and 38 proteins that were downregulated. Furthermore, 103 proteins were intersected with proteins in the autophagy database, and six proteins were found to be related to autophagy. Among them, S100a9 was the most significantly upregulated, suggesting that β_1_-AA-induced upregulation of S100a9 might be involved in myocardial autophagy inhibition. S100a9 is a member of calcium-binding proteins. It belongs to the S100 family and is abundantly expressed in neutrophils and monocytes under pathological conditions [33, 34]. Cardiomyocytes can also produce a large amount of S100a9 protein during myocardial ischemia, myocardial hypertrophy, or when stimulated by inflammation [17, 35–38]. Researchers have found that S100a9 is overexpressed in the myocardium of cardiac infarction and ischemia–reperfusion patients, which was related to the poor prognosis of patients [1, 17, 33, 35, 39–41]. Additionally, S100a9 also plays an important role in autophagy. In human breast cancer cells, S100a9 could promote autophagy by upregulating the Beclin-1 and promoting the formation of Atg12-Atg5 and lysosome activation, thereby inducing cell death [22, 39, 42, 43]. However, in pancreatic cancer cells, the main effects of S100a9 are the inhibition of NF-κB and stimulation of mTOR, both of which inhibit autophagy [23]. In colon cancer cells, S100a9 is oxidized by NOX1-produced ROS, which facilitates binding to mTORC1 and its activation. mTORC1 inhibits autophagy by binding to the ULK1 complex [24]. Based on an analysis of the scRNA-seq and mRNA-seq data of myocardial infarction mice, it has been found that S100a9 regulated autophagy through the MAPK and PI3K-AKT signaling pathways [25]. In this study, we reported that S100a9 was upregulated to suppress autophagy in the presence of β_1_-AA. We speculate that these opposing findings may be due to the different location of S100a9. Research indicates that S100a9 is found in both the intracellular and extracellular spaces, and therefore could have intracellular and extracellular effects [17]. In human breast cancer cells, exogenous S100a9 promotes autophagy through the cell-membrane receptors TLR4 and RAGE. However, in pancreatic cancer cells and colon cancer cells, S100a9 is located in the intracellular space and exerts an inhibitory effect on autophagy, which is similar to what was found in our research. Research has also shown that short-term blocking with a S100a9 blocker (ABR-238901) can reduce cardiac inflammation, limit myocardial damage, and improve the cardiac function of mice with cardiac infarction [18, 35]. However, long-term S100a9 blockade negatively affects cardiac recovery in a murine myocardial infarction model [18]. ABR-238901, the S100a9 inhibitor used in the above study, can prevent S100a9 in the extracellular space from binding to TLR4 and RAGE receptors on the cell membrane. This mechanism of S100a9 blockade is different from that in our study. We focused on the role of S100a9 in the intracellular space and how it exerts an inhibitory effect on autophagy. Therefore, we believe that S100a9 has different effects on autophagy inside and outside the cell. Further, knocking-down S100a9 expression using siRNAs could significantly improve β_1_-AA-induced autophagy inhibition, suggesting that enhanced expression of S100a9 was an important reason for β_1_-AA-induced autophagy inhibition in cardiomyocytes. However, it remains unclear how the upregulation of S100a9 inhibits cardiomyocyte autophagy.

To explore the potential mechanism of S100a9-induced decreased myocardial autophagic flux, an autophagy PCR array was performed to screen the mRNA spectrum of cardiomyocytes after knockdown of S100a9. The tests revealed a significant upregulation of 22 genes and downregulation of 8 autophagic genes. Among them, Atg9a was upregulated significantly. Atg9a is the only transmembrane core autophagy protein, and it plays a key role in the initiation of autophagosome formation [44]. During autophagy, Atg9a is phosphorylated by the ULK1 complex, and phosphorylated Atg9a can recruit LC3 and WIPI1/2 to the autophagosome formation site. At the same time, Atg9a promotes the expansion and extension of autophagosomes [45, 46]. Therefore, Atg9a is a key protein during the early stage of autophagy. Research has shown that inhibition of Atg9a, and thus also of autophagy, was suppressed, which could significantly reverse angiotensin II-induced myocardial hypertrophy [47]. Thus, decreased Atg9a expression suppressing autophagy plays an important role in cardiac dysfunction. In addition, there are genes other than those related to autophagy that may have changed expression in S100a9 knockdown cardiomyocytes. A research has confirmed that knockdown of S100a9 upregulates the expression of myocardial hypertrophy-related genes, such as ANP and β-MHC, in cardiomyocytes [38]. However, our research was focused on the cardiac dysfunction caused by autophagy inhibition, and we thus aimed to confirm which autophagy-related genes contributed to cardiac dysfunction induced by β1-AAs. Therefore, we used a PCR array related to autophagy. In this study, we showed that Atg9a were decreased after stimulation with β_1_-AAs in cardiomyocytes. Knockdown of S100a9 significantly alleviated the Atg9a decrease induced by β_1_-AAs. To further confirm the role of Atg9a in autophagy suppression induced by S100a9 overexpression, we transfected an S100a9 plasmid and an Atg9a plasmid in cardiomyocytes. As expected, overexpression of Atg9a reversed the suppression of autophagy activity caused by S100a9 overexpression. Together, these data indicated that β_1_-AA-induced upregulation of S100a9 could induce the decline of autophagy via inhibiting Atg9a transcription in cardiomyocytes; however, the specific mechanism remained unclear.

Dysfunction of transcription factors is an important mechanism that causes transcriptional inhibition of genes. We identified HIF-1α as an important transcription factor of Atg9a through screening the transcription factor-related databases (JASPAR and Animal TFDB3.0). It has been shown that HIF-1α could bind to the promoter of Atg9a and promote Atg9a transcription [48]. Therefore, HIF-1α was chosen for use in our present study. HIF-1 is composed of α and β subunits, and the subunits of HIF-1α mainly determined its activity, which can regulate oxygen homeostasis. It plays a key role in the pathological and physiological processes [49, 50]. HIF-1α plays a protective role in various cardiovascular diseases, such as ischemic reperfusion and heart failure. It can regulate blood oxygen utilization, glucose metabolism, angiogenesis, tissue remodeling, and other processes [51–53]. The lack of HIF-1α can accelerate the progression of heart failure caused by stress overload, and increasing HIF-1α expression can enhance myocardial autophagy and delay heart failure [54, 55]. Our research found that HIF-1α could significantly increase the luciferase activity of Atg9a in cardiomyocytes. In addition, the results indicated that HIF-1α overexpression could upregulate the level of Atg9a in cardiomyocytes. This suggested that HIF-1α induced Atg9a expression by acting as a transcription factor. Therefore, we speculated that β_1_-AA-induced inhibition of autophagy in cardiomyocytes should be accompanied by the downregulation of HIF-1α expression. However, our study found that β_1_-AAs could increase HIF-1α expression in cardiomyocytes, but the Atg9a luciferase activity was decreased significantly after β_1_-AA treatment, suggesting that β_1_-AAs interfered with the transcriptional activity of HIF-1α. Regarding the issue of β1-AA-induced upregulation of HIF-1α protein levels, a previous study showed that β-AR agonists could transactivate EGFR and elicit Akt and ERK1/2 activity in a PKA-dependent manner, which, in turn, upregulated the levels of HIF-1α and downstream target genes [56]. β1-AA is an agonist of β1-AR, so it may also upregulate HIF-1α protein levels. In general, HIF-1α is regulated by post-translational mechanisms through PHD (prolyl hydroxylase), while HIF-1α protein degradation is regulated by oxygen-dependent prolyl hydroxylation, which targets the protein for ubiquitylation through E3 ubiquitin-protein ligases. These ligases contain the von Hippel-Lindau tumor-suppressor protein (VHL), which binds specifically to hydroxylated HIF-1α [57]. A research has found that PHD activity is specifically upregulated by β2-AR, but not β1-AR, stimulation [58]. Although we found that β_1_-AAs could directly bind to β_1_-AR but not to β_2_-AR [59], we also discovered that β2-AR is involved in the regulation of β1-AR signaling [12]. Therefore, it is still unclear whether β1-AAs affects HIF-1α through PHD activity, which is still a question worth exploring. It is still unclear why increased HIF-1α failed to promote Atg9a transcription.

Normal nuclear entry of HIF-1α is an important prerequisite for its role as a transcription factor [60]. Nuclear and cytoplasmic separation and immunofluorescence staining were used to observe the localization of HIF-1α in cardiomyocytes after β_1_-AA treatment. The results showed that β_1_-AA treatment increased HIF-1α accumulation in the cytoplasm but decreased HIF-1α expression in the nucleus, suggesting that β_1_-AAs inhibited HIF-1α from entering the nucleus normally, thereby decreasing Atg9a transcription. Moreover, we speculated that increased S100a9 might play a role in hindering entry of HIF-1α into the nucleus. Using immunofluorescence staining and a Co-IP assay, we observed a cytoplasmic distribution of HIF-1α and significantly increased binding with S100a9 upon β_1_-AA stimulation. S100a9 can form a complex with not only HIF-1α, but also with other transcription factors. The transcription factors that S100a9 can bind to deserve further research. Even if we do not find other transcription factors that can bind to S100a9, the findings of this study remain unaffected; that is, S100a9 can bind to HIF-1α to affect autophagy in myocardial cells.

**Fig. 7:**
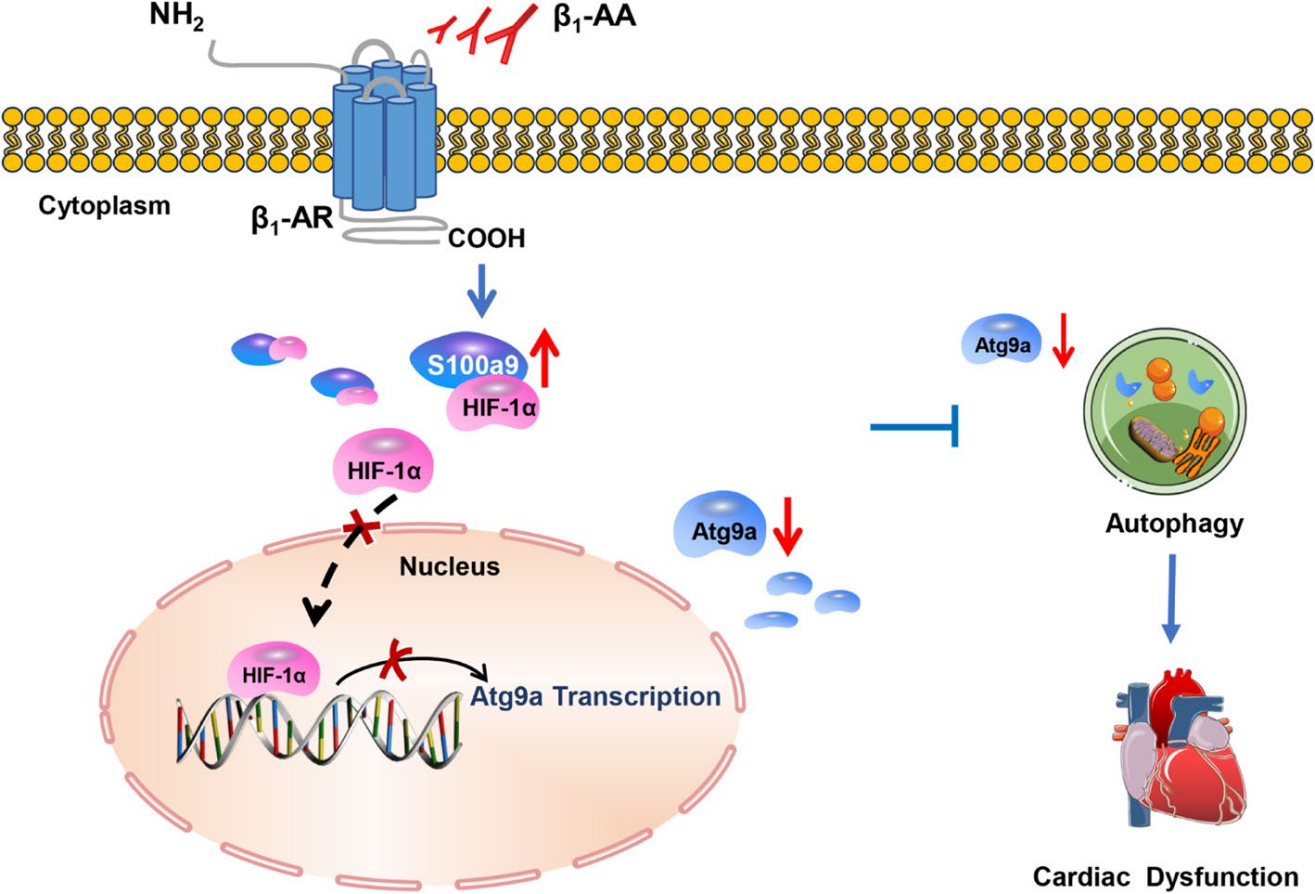
Working model showing that β_1_-AAs increase the expression of S100a9 in cardiomyocytes. Enhanced binding of S100a9 and HIF-1α in the cytoplasm results in decreased entry of HIF-1α into the nucleus. This inhibits expression of Atg9a, leading to decreased myocardial autophagy flux, thereby causing cardiac dysfunction

Therefore, we believed that β_1_-AAs induced the upregulation of S100a9 expression in cardiomyocytes. The resulting increased expression of S100a9 inhibited Atg9a expression by competitively binding to HIF-1α and preventing HIF-1α translocation into the nucleus, thus inhibiting autophagy and promoting cardiac dysfunction (Fig. 7). Furthermore, we used AAV9-S100a9-RNAi adeno-associated virus to knockdown S100a9 in mouse cardiomyocytes and found that it could significantly improve β_1_-AA-induced autophagy inhibition and cardiac dysfunction. In one study, it was found that β1-AR activation was responsible for heart failure in 38% of patients with heart failure [61]. Overactivation of β1-AR in cardiomyocytes is a core heart failure mechanism [62]. Our research group, as well as others, have shown that in addition to the endogenous β1-AR ligand norepinephrine (NE), autoantibodies against β1-AR (β1-AA), with agonist-like effects, circulate in the sera of 40–60% of heart failure patients [7, 63]. In this study, we found that stimulation of β1-AR by β1-AA increased the expression of S100a9 and decreased myocardial autophagy. Other studies have shown that the β-AR agonist ISO also increased S100a9 expression in myocardial injury [64], and autophagy activity was also significantly reduced in myocardial hypertrophy induced by ISO [65, 66]. In addition, a previous study showed that atenolol, a selective β_1_-adrenergic receptor antagonist, partially inhibited the expression of S100a9 [67]. Our research group indicated that the β_1_-AR antagonist atenolol could partially reverse the decline of autophagy induced by β_1_-AAs in cardiomyocytes [13]. Therefore, we speculated that in heart failure caused by β1-AR activation, the upregulation of S100a9 causes autophagy inhibition. However, this mechanism needs further confirmation in heart failure induced by other factors. Therefore, downregulation of S100a9 can be used as an effective strategy to upregulate myocardial autophagy and improve cardiac dysfunction. Through the above research, we tried to identify new targets for the precise regulation of myocardial autophagy, thereby providing a new theoretical basis for the precise treatment of cardiovascular disease.

## Conclusion

β_1_-AA inhibited autophagy by inducing upregulation of S100a9 expression in cardiomyocytes. Increased S100a9 inhibits Atg9a expression by competitively binding to the transcription factor HIF-1α to block its entry into the nucleus, thereby inhibiting autophagy and leading to cardiac dysfunction.

### Supplementary Information

**Additional file 1. Tables S1–S3** and **Figs. S1–S7**.

**Additional file 2. **Raw data of PCR Array.

**Additional file 3. **Echocardiography values.

## Data Availability

All data generated or analyzed during this study are included in this article (and its additional files).

## Abbreviations

β_1_-AAs: β_1_-Adrenergic receptor autoantibodies
β_1_-AR: β_1_-Adrenergic receptor
ECII: Second extracellular loop
SA: Streptavidin
ABTS: 2, 2′-Azinodi (ethylbenzthiazoline) sulfuric salt
TMT: Tandem mass tag
NC: Negative control
DMEM: Dulbecco’s Modified Eagle Medium
FBS: Fetal bovine serum
HPLC: High-performance liquid chromatography
DEPs: Differentially expressed proteins
RIPA: Radioimmunoprecipitation assay
Co-IP: Co-immunoprecipitation
BCA: Bicinchoninic acid
HIF-1α: Hypoxia inducible factor-1α
siRNA: Small-interfering RNA
PCA: Principal component analysis
FC: Fold change
AAV9: Adeno-associated virus 9
EF%: Ejection fraction
FS%: Fractional shortening
LVIDd: Left ventricular end-diastolic diameter
LVIDs: Left ventricular end-systolic diameter
IVSd: Interventricular septal thickness at diastole
IVSs: Interventricular septal thickness at systole
