# Supplementary material for: S100a9 inhibits Atg9a transcription and participates in suppression of autophagy in cardiomyocytes induced by β1-adrenoceptor autoantibodies
Source: Cell Mol Biol Lett. 2023 Sep 18;28:74. doi: 10.1186/s11658-023-00486-1 (PMC10506287; doi:10.1186/s11658-023-00486-1)
Supplement: Supplementary file 1 — Additional file 1. Tables S1–S3 and Figs. S1–S7. [file 11658_2023_486_MOESM1_ESM.docx]

Tables

Table S1. Number of intersections between statistically significant proteins in proteomics and autophagy datasets.

| Number | Accession | Protein description | Gene name | FC |
| --- | --- | --- | --- | --- |
| 1 | P31725 | Protein S100-a9 | S100a9 | 1.76 |
| 2 | Q9CY18 | Sorting nexin-7 | Snx7 | 1.375 |
| 3 | Q61696 | Heat shock 70 kDa protein 1A | Hspa1a | 1.212 |
| 4 | P70347 | TRAF family member-associated NF-kappa-B activator | Tank | 1.202 |
| 5 | P24270 | Catalase | Cat | 0.813 |
| 6 | Q8BGT0 | Osteopetrosis-associated transmembrane protein 1 | Ostm1 | 0.377 |

Table S2. Small-interfering RNA (siRNA) sequences.

| Number | Name | Sequence |
| --- | --- | --- |
| 1 | siRNA NC | F:UUCUCCGAACGUGUCACGU TT;  R:ACGUGACACGUUCGGAGAA TT |
| 2 | S100a9 (r) -si-1 | F:GGAGAAAAGAAAUGAAAAU TT;  R:AUUUUCAUUUCUUUUCUCC TT |
| 3 | S100a9 (r) -si-2 | F:GAUGCUGAUGGGAAAGUUGAU TT;  R:AUCAACUUUCCCAUCAGCAUC TT |
| 4 | S100a9 (r) -si-3 | F:GAAAUAAUUUAAAAAUGAA TT;  R:UUCAUUUUUAAAUUAUUUC TT |

Table S3. Primer sequences.

| Number | Name | Sequence |
| --- | --- | --- |
| 1 | S100a9 | F: 5′-AGACATCATGGAGGACCTGGACAC-3′;  R: 5′-TGGGTTCTCATGCAGCTTCTC-3′ |
| 2 | LC3II | F: 5′-AGCTCTGAAGGCAACAGCAACA-3′;  R: 5′-GCTCCATGCAGGTAGCAGGAA-3′ |
| 3 | GAPDH | F: 5′-GGCACAGTCAAGGCTGAGAATG-3′;  R: 5′-ATGGTGGTGAAGACGCCAGTA-3′ |
| 4 | Atg9a | F: 5′-CAAGCCCGCCTCCAAGTACATG-3′;  R: 5′-TCCACAGCCAACACATCTTCATCG-3′ |

Figures


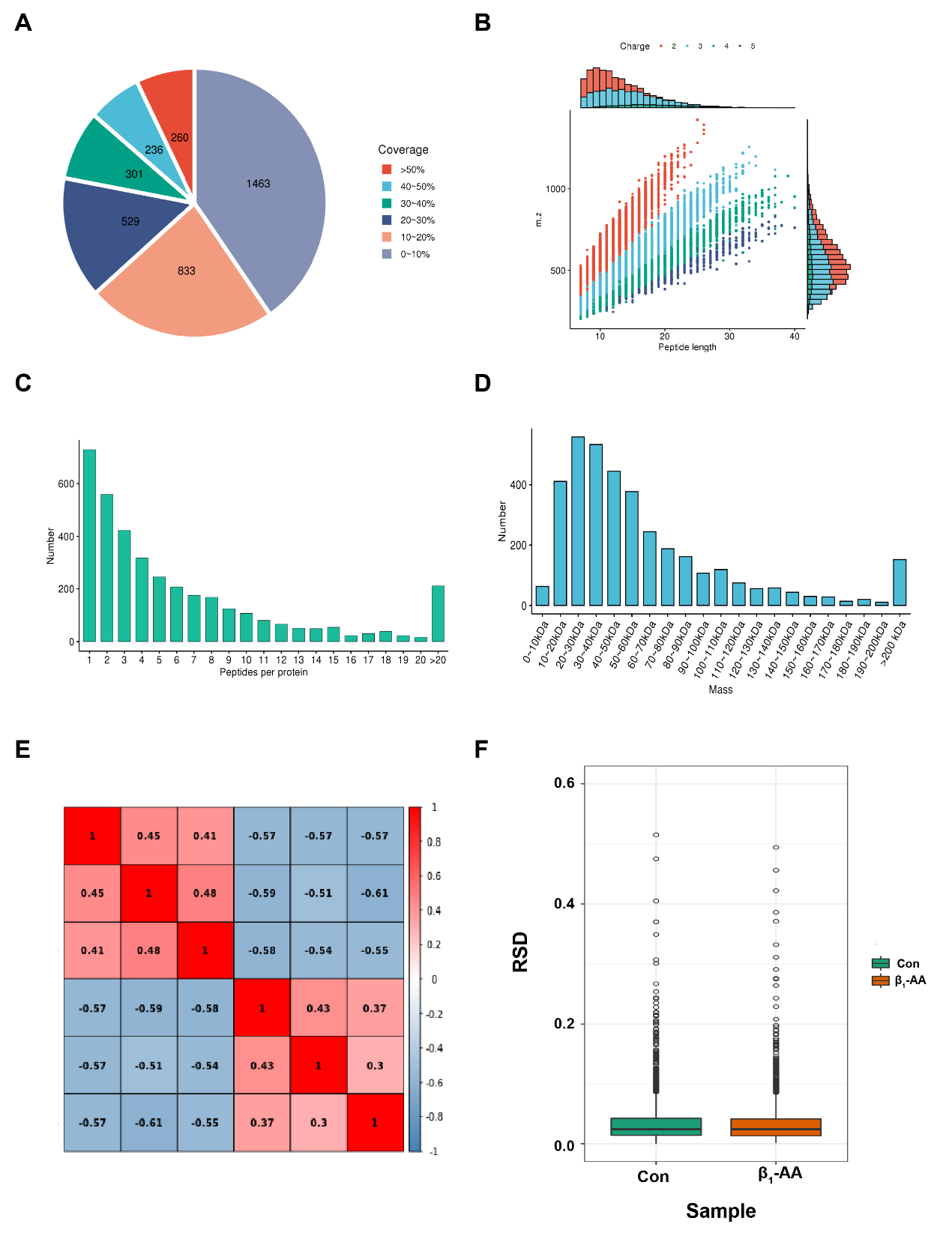


Figure S1. Quality control of the tandem mass tag (TMT)-based quantitative proteomics analysis.

A) Coverage distribution of proteins identified by mass spectrometry. B) Length distribution of proteins using the quantified proteins. C) Distribution of peptide number; X: protein peptide number, Y: number of proteins identified by mass spectrum. D) Molecular weight distribution of the proteins identified; X: protein molecular weight, Y: the number of proteins. E) Heat map of the pairwise calculation of Pearson’s correlation coefficients of all samples. As a value measuring the linear correlation degree of two groups’ data, when the Pearson’s coefficient is closer to −1, it indicates a negative correlation; when it is closer to 1, it indicates a positive correlation, and when it is close to 0, it indicates no correlation. F) Box plots of the relative standard deviation (RSD) distribution of the repeated samples using the quantified proteins. The smaller
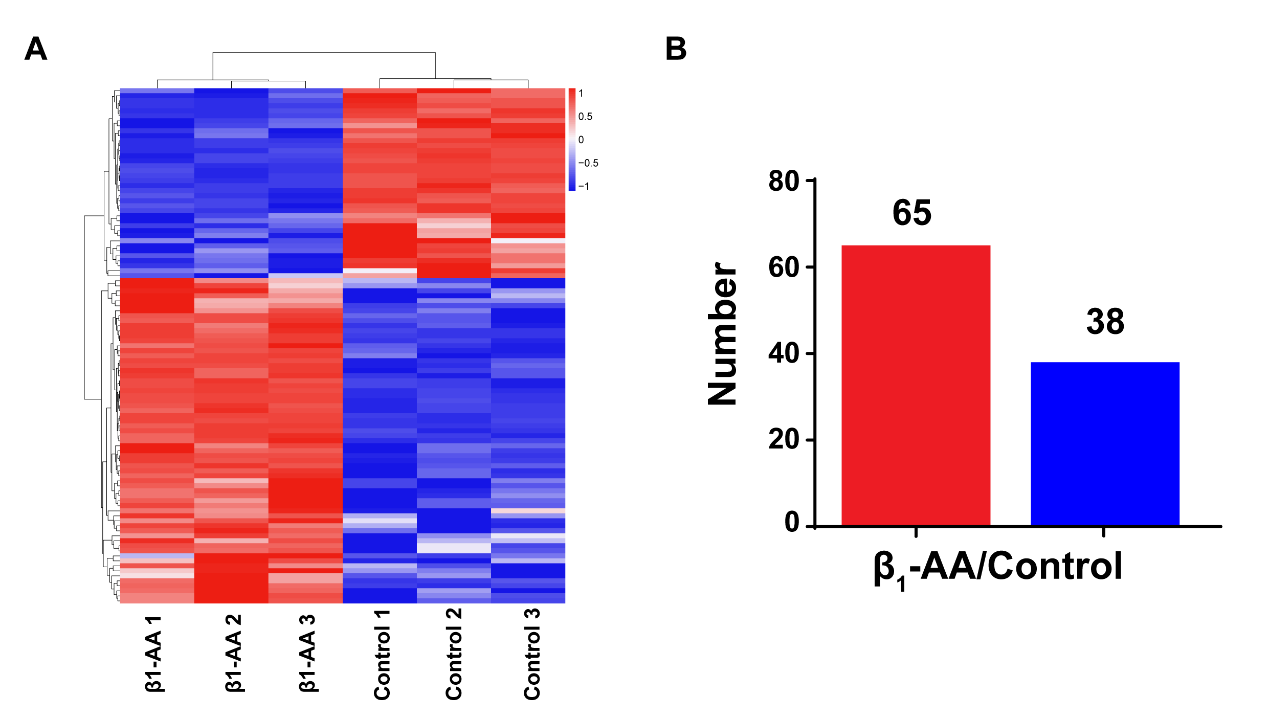
the overall RSD value, the better the quantitative repeatability.

Figure S2. Proteomics analysis and protein quantification.

A) Quantitative heatmap of differentially expressed proteins in the myocardium of mice. Red represents upregulated proteins and blue represents downregulated proteins. B) Column diagram of the numerical distribution of differentially expressed proteins.


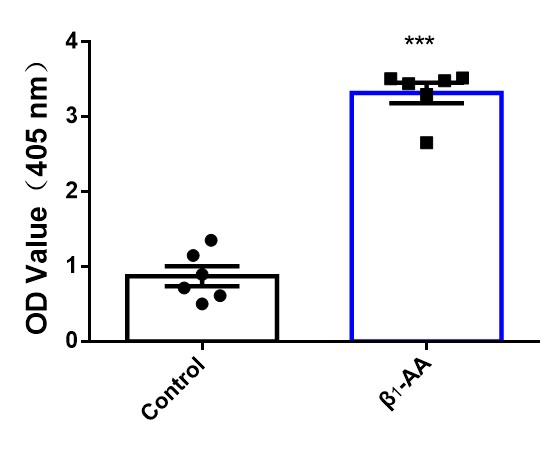


Figure S3. OD values of serum β_1_-AAs in mice after active immunization for 4 weeks. *** p < 0.001 vs. control.


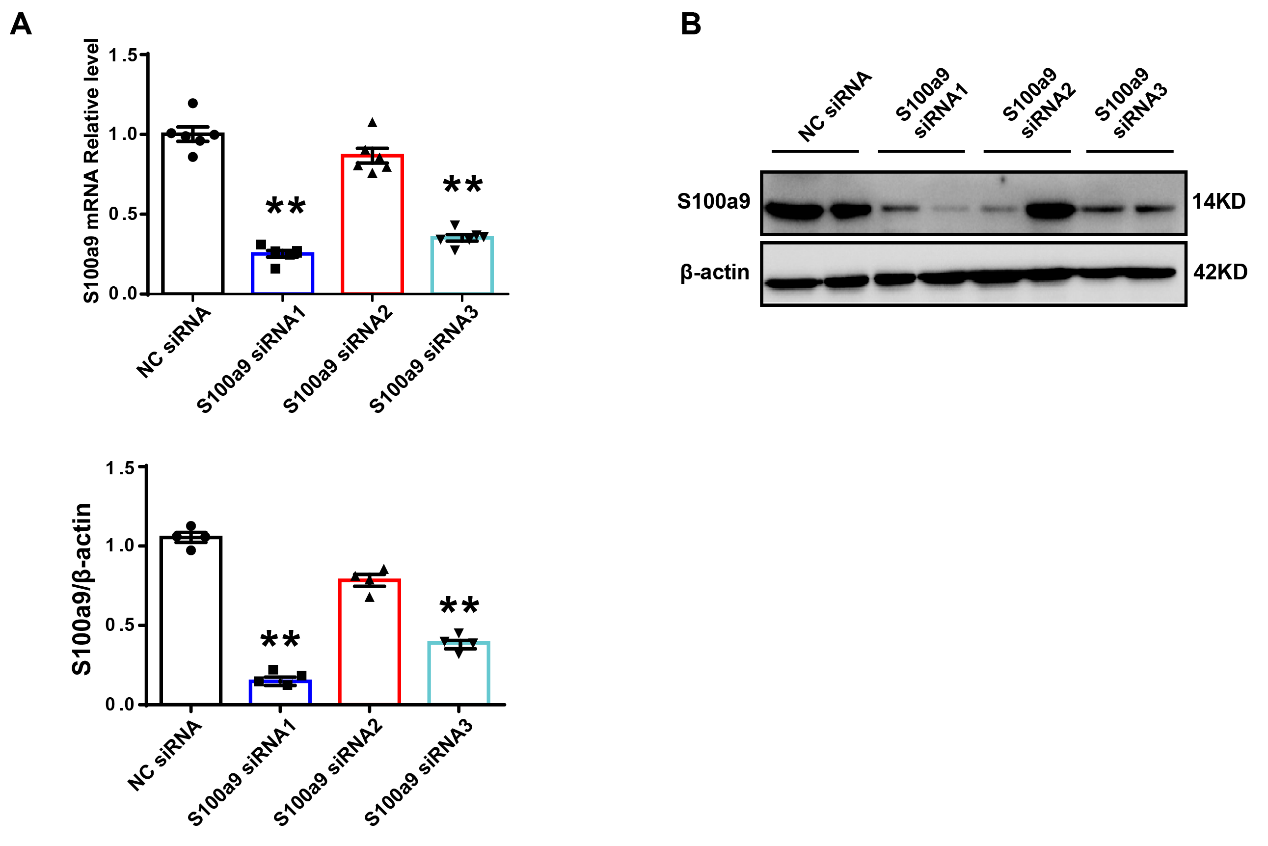


Figure S4. Knockdown effect of S100a9 in cardiomyocytes.

A) RT-PCR was used to determine the expression level of S100a9 in H9c2 cells after transfection with S100a9 siRNA or NC siRNA. B) Western blot analysis of S100a9 in H9c2 cells after transfection with S100a9 siRNA or NC siRNA. Data are presented as the mean ± SEM (n = 4 per group); * p < 0.05 vs. control, ** p < 0.01 vs. control.


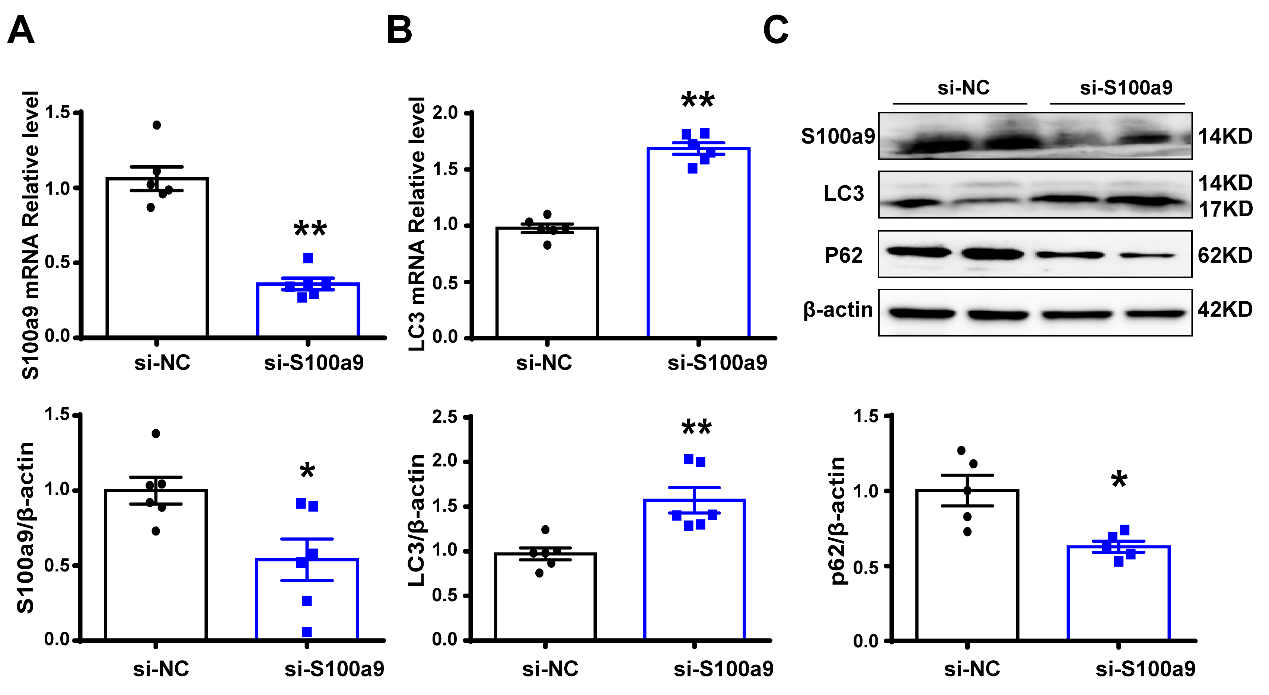


Figure S5. Knockdown of S100a9 increased autophagy levels in cardiomyocytes.

A–B) RT-PCR was used to determine the expression level of S100a9 and LC3 in H9c2 cells after transfection with S100a9 siRNA or NC siRNA for 36 h. C) Western blot analysis of S100a9, LC3, and p62 in H9c2 cells after transfection with S100a9 siRNA or NC siRNA for 36 h. Data are presented as the mean ± SEM (n = 6 per group); * p < 0.05 vs. control, ** p < 0.01 vs. control.


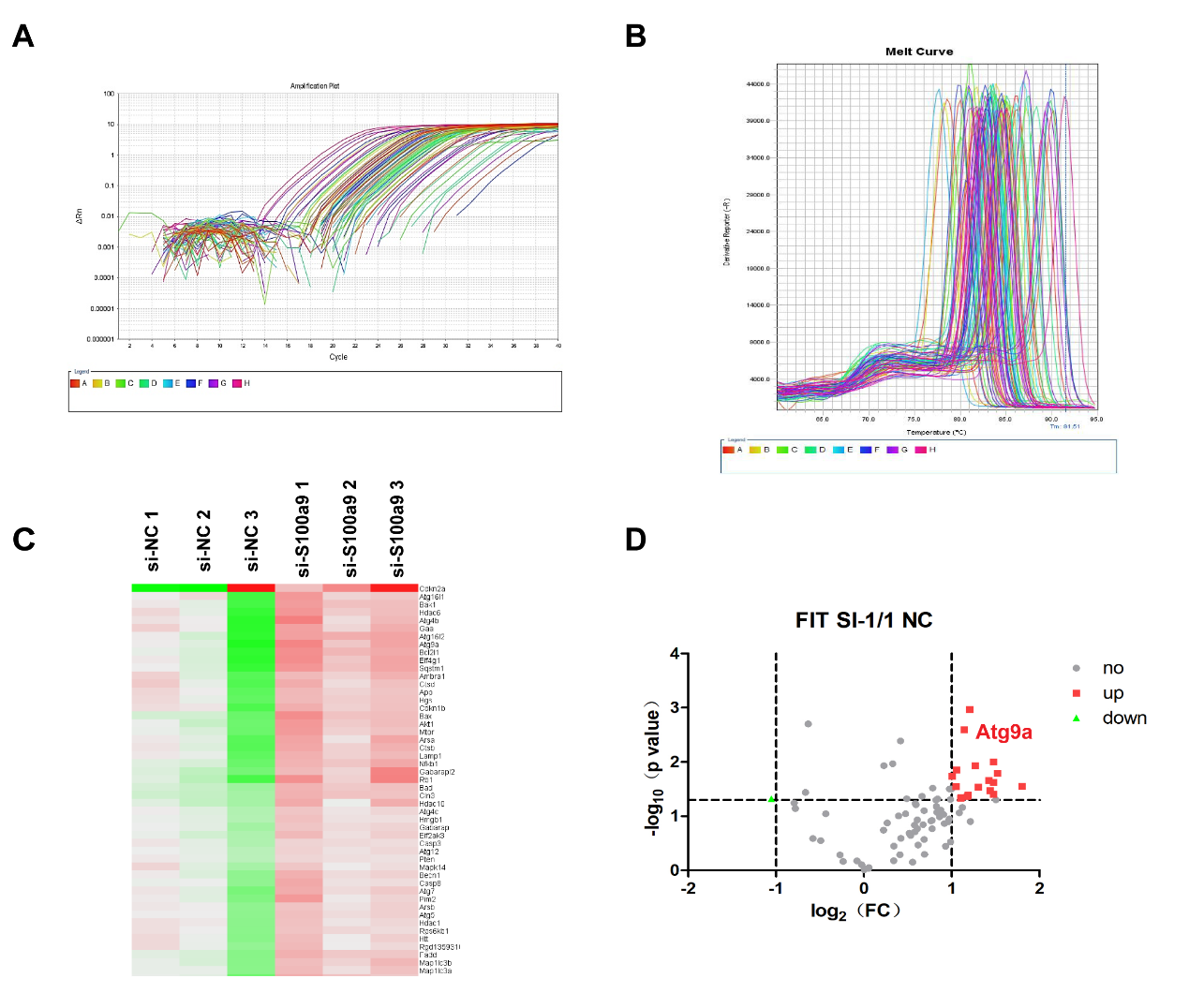


Figure S6. PCR array results showed changes in relative Atg9a levels in cardiomyocytes transfected with S100a9 siRNA.

A) Fluorescence quantitative PCR dissolution curve. B) Fluorescence quantitative PCR amplification curve. C) Heatmap of quantitative differentially expressed genes in cardiomyocytes transfected with S100a9 siRNA and NC siRNA. D) Volcano plot of the differentially expressed genes.


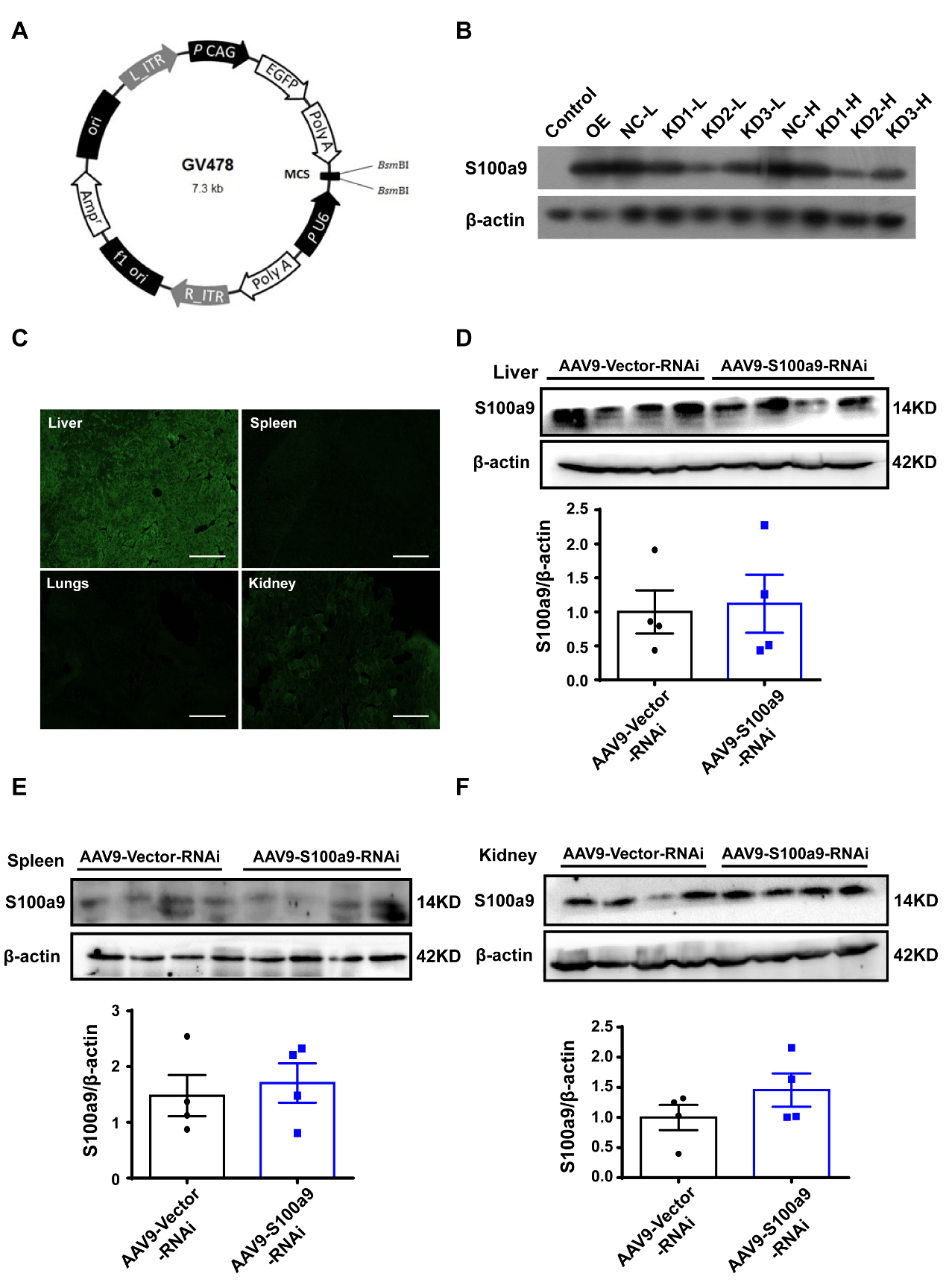


Figure S7. Construction of an AAV9-S100a9-RNAi adeno-associated virus vector and verification of effective RNAi targets by external screening.

A) AAV9-S100a9-RNAi adeno-associated virus vector. B) Effective target of S100a9 was detected by western blot. C) Fluorescence detection of virus enrichment in frozen liver, spleen, lung, and kidney sections. D–F) Western blot was used to detect the knockdown effect of S100a9 in liver, spleen, and kidney. Data are presented as the mean ± SEM (n = 4 per group). Control: cell group without transfection of any plasmid (empty cell group); NC: co-transfection of overexpression plasmid and negative control adeno-associated virus cell group (negative control group); KD: co-transfection of overexpression plasmid and RNAi adeno-associated virus cell group (knockdown group); OE: Overexpression, L: low, H: high.
